# Submicroscopic Gametocytes and the Transmission of Antifolate-Resistant *Plasmodium falciparum* in Western Kenya

**DOI:** 10.1371/journal.pone.0004364

**Published:** 2009-02-05

**Authors:** Mayke J. A. M. Oesterholt, Michael Alifrangis, Colin J. Sutherland, Sabah A. Omar, Patrick Sawa, Christina Howitt, Louis C. Gouagna, Robert W. Sauerwein, Teun Bousema

**Affiliations:** 1 Department of Medical Microbiology, Radboud University Nijmegen Medical Centre, Nijmegen, The Netherlands; 2 Centre for Medical Parasitology at the Department of International Health, Immunology and Microbiology, University of Copenhagen, and at the Department of infectious Diseases, Copenhagen University Hospital (Rigshospitalet), Copenhagen, Denmark; 3 Department of Infectious and Tropical Diseases, London School of Hygiene and Tropical Medicine, London, United Kingdom; 4 Kenya Medical Research Institute, Centre for Biotechnology Research and Development, Nairobi, Kenya; 5 Human Health Division, International Centre of Insect Physiology and Ecology, Mbita, Kenya; 6 Institut de Recherche pour le Développement, Bobo Dioulasso, Burkina Faso; Mahidol University, Thailand

## Abstract

**Background:**

Single nucleotide polymorphisms (SNPs) in the *dhfr* and *dhps* genes are associated with sulphadoxine-pyrimethamine (SP) treatment failure and gametocyte carriage. This may result in enhanced transmission of mutant malaria parasites, as previously shown for chloroquine resistant parasites. In the present study, we determine the association between parasite mutations, submicroscopic *P. falciparum* gametocytemia and malaria transmission to mosquitoes.

**Methodology/Principal Findings:**

Samples from children treated with SP alone or in combination with artesunate (AS) or amodiaquine were genotyped for SNPs in the *dhfr* and *dhps* genes. Gametocytemia was determined by microscopy and *Pfs25* RNA–based quantitative nucleic acid sequence–based amplification (*Pfs25* QT-NASBA). Transmission was determined by membrane-feeding assays. We observed no wild type infections, 66.5% (127/191) of the infections expressed mutations at all three *dhfr* codons prior to treatment. The presence of all three mutations was not related to higher *Pfs25* QT-NASBA gametocyte prevalence or density during follow-up, compared to double mutant infections. The proportion of infected mosquitoes or oocyst burden was also not related to the number of mutations. Addition of AS to SP reduced gametocytemia and malaria transmission during follow-up.

**Conclusions/Significance:**

In our study population where all infections had at least a double mutation in the *dhfr* gene, additional mutations were not related to increased submicroscopic gametocytemia or enhanced malaria transmission. The absence of wild-type infections is likely to have reduced our power to detect differences. Our data further support the use of ACT to reduce the transmission of drug-resistant malaria parasites.

## Introduction

Artemisinin-based combination therapy (ACT) is now widely advocated as first-line antimalarial treatment. Nevertheless, drugs that are now no longer recommended as first-line treatment, such as sulphadoxine-pyrimethamine (SP), will continue to play a role in malaria control. A change in international therapeutic recommendations is not always translated into an immediate, effective policy change at country levels. In Kenya, monotherapy with chloroquine was replaced by SP in 1998 and SP was officially replaced by the ACT Artemether-Lumefantrine (AL) in 2004 [1]. However, health worker training and distribution of AL were not completed until the end of 2006 [1]. Even if official recommendations are followed by government services, monotherapy will remain available in the private sector, as was reported in Nigeria [2]. Furthermore, SP also plays an important role in intermittent preventive treatment to control malaria in infants [3], [4] and pregnant women [5] and in some situations the combination of SP with amodiaquine (AQ) may be a cheap and efficacious alternative to ACT [6], [7]. The ongoing use of SP, either in treatment of uncomplicated malaria or as method of malaria control by intermittent preventive treatment, underlines the importance of continued studies of SP resistance and the way resistance spreads in populations.

SP resistance is associated with the presence of single nucleotide polymorphisms (SNPs) in the *dhfr* and *dhps* genes [8]–[10]. This SP resistance starts off with a core SNP at codon 108 (S108N), which is followed by mutations at c51 (N51I) and 59 (C59R) [11] leading to a so-called ‘triple mutation’. Additional mutations in the *dhps* gene (codon 437 and 540) are related with even higher risks for SP treatment failure [12]. Other codons (in *dhfr* at c164 and *dhps* at c581 and c613) are also associated with high-level SP resistance in Southeast Asia [13], [14] and Latin America [9], but until now, these mutations are uncommon on the African continent and have rarely been found in East Africa [15]–[19].

The presence of mutations in the *dhfr* and *dhps* genes may not only predict treatment outcome, but may also be related to the transmission stages of *P. falciparum*, the gametocytes. The presence of resistant parasites at enrolment was found to be related to microscopically detected gametocytes during follow-up [20], [21] or even prior to treatment [21] and to the duration of gametocyte carriage [22]. Mutations in the *Pfcrt* and *Pfmdr1* genes, encoding CQ resistance, were associated with a higher gametocyte density and subsequent malaria transmission after CQ monotherapy [23], [24] or SP/CQ combination treatment [25]. Recently, a study of Méndez and others showed that in Colombia, malaria transmission was enhanced for parasites with low levels of resistance to SP compared to fully sensitive parasites [26]. In addition to a transmission advantage of mutant parasites in the human host through a higher gametocyte prevalence or density [20], [23], there may also be a selective advantage in the mosquito midgut, as was suggested for *Pfcrt* mutations [23]. These findings are worrying, indicating a very rapid spread of mutant parasites under drug pressure, but need confirmation in areas with different levels of transmission intensity and drug resistance.

Molecular gametocyte detection tools have recently demonstrated that gametocyte carriage can be seriously underestimated by the use of microscopy [27]. Submicroscopic gametocytes are not only common in various populations [27]–[29], but may also contribute considerably to malaria transmission [27].

The present study was conducted in an area of high resistance to SP with more than 50% treatment failure within one month of follow-up [27]. Samples from a previously published study on submicroscopic gametocytemia following antimalarial treatment [27] were genotyped to determine the relation between parasite mutations, submicroscopic gametocytemia and the transmission of malaria to mosquitoes.

## Methods

### Ethics statement

The study protocol (SSC No. 791) was approved by the Scientific Steering Committee and Ethical Review Committee of the Kenya Medical Research Institute. Written, informed consent was obtained from a parent or guardian of the participating children. The trial was completed in 2004, before registration became mandatory for clinical trials. The trial was registered after completion under Clinical Trials registration number ISRCTN31291803 (http://www.controlled-trials.com/ISRCTN31291803).

### Objectives

We hypothesized that mutant parasites possess a transmission advantage compared to susceptible parasites. To test this hypothesis, our primary objectives were to determine the number of allelic variants before and after treatment and to determine the relation between mutations in the *dhfr* and *dhps* genes and: i) gametocyte prevalence and density by microscopy and RNA-based quantitative nucleic acid sequence based amplification (QT-NASBA); ii) mosquito infection rates; iii) oocyst densities in mosquitoes.

### Participants

This study was conducted from October to December 2004 (short rainy season) in Mbita, a rural village on the shores of Lake Victoria in Suba District, western Kenya. The main malaria vectors in the area are *A. gambiae*, *A. funestus*, and *A. arabiensis*. Malaria transmission is high and perennial, with parasite prevalences in the human population ranging from 24.4% to 99.0%. Generally, the rainfall pattern is bimodal, with a long rainy season between March and May and a short rainy season between October and December.

The study population and treatment procedures are described elsewhere [27]. Briefly, 528 children (6 months–10 years) with *P. falciparum* mono-infection with a minimum parasite density of 500/parasites µL, fever or history of fever in the last 48 hours and absence of danger signs were enrolled and randomised to treatment with sulphadoxine-pyrimethamine (SP; Fansidar®, Roche, Switzerland), SP plus amodiaquine (AQ; Camoquine®, Pfizer, Senegal), SP + Artesunate (AS; Arsumax®, Sanofi, France) or Artemether-Lumefantrine (AL; CoArtem®, Novartis Pharma, Switzerland). Samples were randomly selected from the study populations of 2003 and 2004 for molecular analysis of gametocyte carriage [27]. In addition, a random selection of 25 children per treatment arm was invited for membrane feeding experiments and molecular analysis of gametocyte carriage, irrespective of gametocyte carriage status [27]. Samples from these two groups were combined and used for parasite genotyping in the current study. Only children who received SP alone or in combination with AQ or AS were included in the current study because we were interested in the transmission potential in relation to mutations in the *dhfr* and *dhps* genes (i.e. mutations that are associated with SP treatment failure). In addition, we determined the prevalence of mutations in the *Pfcrt* gene that are associated with AQ treatment failure.

### Description of procedures

#### Microscopy and Pfs25 QT-NASBA

Blood smears were stained with 10% Giemsa for 10 min and then screened for asexual parasites and gametocytes at enrolment and on days 3, 7, 14, and 28. Asexual parasites and gametocytes were counted against 200 and 500 white blood cells (WBCs), respectively, and the counts were converted to parasites per microliter on the assumption of a density of 8000 WBCs/µL. Parasite detection by *Pfs25* QT-NASBA was done at day 0, 3, 7, 14 and 28 as described elsewhere [30], [31], using a NucliSens EasyQ analyser (bioMérieux) as described elsewhere for *Pfs25* mRNA [30]. Nucleic acids were extracted from 50-µL blood samples as described by Boom and others [32]. The *Pfs25* QT-NASBA technique is gametocyte specific and has a detection limit of 0.02–0.1 gametocytes/µL. NucliSens Basic kits were used for amplification, in accordance with the manufacturer's instructions. A standard dilution series of mature, in vitro–cultured NF54 gametocytes [33] was included in each run to determine gametocyte densities in the test samples.

#### Mosquito membrane feeding and dissection

Membrane-feeding assays were conducted on day 14 after treatment, as described elsewhere [27]. Briefly, 3-mL venous blood samples were obtained and fed to ∼150 locally reared [34] 4–5-day-old female *A. gambiae* sensu stricto mosquitoes via an artificial membrane attached to a water-jacketed glass feeder maintained at 37°C. After 10–15 minutes, fully fed mosquitoes were selected and kept on glucose for 7 days at 27°C–29°C, at which time midguts were dissected in 2% mercurochrome. Midguts were examined microscopically for oocysts, with a second microscopist confirming their presence if observed. Exactly 30 mosquitoes were dissected per child.

#### Detection of SNPs in dhfr/dhps and Pfcrt by PCR and SSOP-ELISA

Samples collected at patient enrolment were screened for SNPs at *dhfr* c108, c59 and c51, using PCR-SSOP as previously described [25], [35] and provided an estimate of baseline allele frequencies at this locus. Samples from individuals participating in membrane feeding experiments were more extensively studied for SNPs in both the *dhfr* and *dhps* genes. For these samples, SNPs at *dhfr* (at c50/51, c59, c108 and c164), *dhps* (at c436/437, c540, c581 and c613) and *Pfcrt* (position 72–76) were determined by SSOP-ELISA of PCR amplified fragments using conditions and reagents as described by Alifrangis and others [36]. For each SNP analyses, parasite samples were categorized into single, mixed but with one SNP in majority or mixed infections with no SNP in majority as described by Alifrangis and others [36]. The mixed samples with one SNP in majority were considered as single mutations. All SSOP-ELISA data were divided into wild or mutant type; samples with a mixed infection (wild-type and mutant) were considered as mutant. Samples with a mutation in c108+c59 or c108+c51 of *dhfr* but no additional mutations were classified as double mutations; samples with mutations in c51+c59+c108 as triple mutations; samples with mutations in c51+c59+c108 of *dhfr* and c437 of *dhps* were considered quadruple mutations; and samples with mutations in c51+c59+c108 of *dhfr* and c437+c540 of *dhps* as quintuple mutations. For confirmation of the rare c164L genotype in *dhfr* the PCR-restriction fragment length polymorphism (RFLP) method described by Duraisingh and others [37] was used.

### Statistical methods and sample size considerations

The major objective of the current study was to determine post-treatment malaria transmission potential in relation to mutations in the *dhfr* and *dhps* genes. This objective was studied using samples from a previously published drug efficacy trial [27] where the sample size for transmission experiments was based on the comparison of transmission potential after SP monotherapy compared to ACT. In the absence of information on the prevalence of mutations in the *dhfr* and *dhps* genes in the study area or their expected association with submicroscopic gametocytes and malaria transmission, we based sample size considerations for the current study on previously published studies on the relation between mutations in the *Pfcrt* gene and malaria transmission. Three previously conducted studies included 11–25 membrane-feeding experiments per treatment arm with an average of 20–30 mosquitoes per experiment [23]–[25]. We included samples from 75 membrane-feeding experiments in randomly selected individuals after SP (n = 25), SP+AQ (n = 25) or SP+AS treatment (n = 25). Thirty mosquitoes were examined per experiment, giving a total of 2250 mosquitoes [27], [38]. Because post-treatment gametocytemia and malaria transmission was previously shown to be similar for SP and SP+AQ [27], data was grouped as non-ACT (SP mono-therapy and SP+AQ combination therapy) or ACT (SP+AS) for analyses on the relation between mutant genotypes and post-treatment gametocytemia and malaria transmission. The transmission potential during follow-up was quantified as the area under the curve of *Pfs25* QT-NASBA gametocyte density versus time (AUC). This measure incorporates both the magnitude and the duration of gametocyte carriage and was described by Mendez and others [39]. Mosquito transmission experiments were analyzed based on the proportion of infectious children, the proportion of infected mosquitoes and oocyst densities in infected mosquitoes.

Statistical analyses were done using SPSS for Windows (version 12.0; SPSS) and Stata (version 8.0; Stata Corporation). Proportions were compared using the χ^2^ test and the McNemar test for matched-pair data was used to analyse the directional selection of mutations from day 0 to day 14. Normally distributed continuous variables were compared using the Student's *t* test. Variables that were not normally distributed were compared using the Wilcoxon rank-sum test. The influence of the number of mutations in infections at enrolment on the proportion of infected mosquitoes at day 14 was tested using logistic regression models with Generalized Estimating Equations (GEE); the influence of the number of mutations in infections at enrolment on oocyst densities in mosquitoes was determined using negative binomial regression. Estimates were adjusted for observations from the same individual. Regression coefficients (β) were calculated with standard errors (se).

## Results

### Prevalence of mutant dhfr genotypes, enrolment parasitemia and gametocyte carriage

The relation between mutations in *dhfr*, enrolment parasitemia, parasite clearance and post-treatment gametocyte carriage was determined in baseline samples from 134 individuals treated with SP or SP+AQ (non-ACT) and 57 individuals treated with SP+AS (ACT). None of the infections consisted solely of wild-type parasites at the *dhfr* locus: all 191samples had the 108N mutation and at least one additional mutation in c51 (85.9%, 164/191) or c59 (79.6%, 152/191). (The I164L substitution at codon 164 was not evaluated in these preliminary experiments.) Based on these three codons, 33.5% (64/191) of the parasite isolates harboured double mutant *dhfr* loci; the remaining 66.5% (127/191) harboured infections with mutations at all three codons. In the absence of wild-type infections as reference category, these two groups were compared.

Enrolment asexual parasite density was not related to the number of *dhfr* mutations (table 1). Asexual parasites were still detectable on day 3 after treatment in 14.5% (19/131) of the individuals treated with non-ACTs and in 1.8% (1/56) of those treated with ACTs (p<0.001), without a relation with the number of parasite mutations. Gametocyte prevalence at enrolment was 26.5% (50/189) by microscopy and 91.4% (118/129) by *Pfs25* QT-NASBA, and was not associated with the number of *dhfr* mutations. Transmission potential during follow-up was quantified as the area under the curve (AUC) of *Pfs25* QT-NASBA gametocyte density versus time for non-ACT and ACT treated children (table 1). The AUC was significantly lower for ACT compared to non-ACT treated children (p<0.001). Within the group of non-ACT treated individuals, AUC was positively associated with the presence of asexual parasites on day three after treatment (i.e. a longer parasite clearance time), p = 0.046. There was no relation between AUC and the presence of three mutations in infections for either non-ACT or ACT treated individuals.

**Table 1:** Enrolment characteristics and gametocyte carriage in relation to mutations in the *dhfr* gene.

	*Double mutation* *	*Three mutations* *	*p-value*
Median asexual parasite density at enrolment (IQR)			
Non-ACT	10,880 (2,280–31,820)	14,320 (5,020–30,360)	0.27
ACT	12,080 (4,560–39,200)	13,560 (4,020–32,760)	0.99
Proportion of parasitemic children at day 3, % (n/N)			
Non-ACT	10.0 (4/40)	16.5 (15/91)	0.33
ACT	4.5 (1/22)	0.0 (0/34)	0.39
Adequate clinical response, % (n/N)			
Non-ACT	58.3 (21/36)	63.6 (49/77)	0.59
ACT	71.4 (15/21)	86.7 (26/30)	0.18
Microscopic gametocyte prevalence at enrolment, % (n/N)			
Non-ACT	22.5 (9/40)	26.1 (24/92)	0.66
ACT	21.7 (5/23)	35.5 (12/34)	0.27
QT-NASBA gametocyte prevalence at enrolment, % (n/N)			
Non-ACT	88.9 (16/18)	91.5 (65/71)	0.66
ACT	87.5 (14/16)	95.8 (23/24)	0.55
Median AUC of QT-NASBA gametocyte density¥/µL versus time¶, (IQR)			
Non-ACT	4.23 (0.39–20.14)	6.13 (1.56–15.88)	0.54
ACT	0.17 (0.02–2.82)	0.29 (0.17–82)	0.35

AUC = area under the curve; GM = geometric mean; IQR = interquartile range; Non-ACT = treatment with sulphadoxine-pyrimethamine (SP) alone or in combination with amodiaquine; ACT = treatment with SP and artesunate (3 days).

¥ Gametocyte densities were measured by *Pfs25* QT-NASBA.

* Mutations were determined at enrolment: double mutation = mutations in c108+c59 or c108+c51; three mutations = mutations in c51+c59+c108;

¶ the period from enrolment until time of feeding (day 14) was considered for determining the AUC.

The lack of wild-type *dhfr* loci among this preliminary baseline analysis at three codons suggested that it would not be possible to find associations between parasite genotype and treatment outcomes of interest such as gametocyte carriage and mosquito infectivity using this approach. Therefore, in subsequent analyses we tested for the (rare) substitution of Leucine for isoleucine at codon 164 of *dhfr,* and performed a full analysis of the *dhps* gene at codons 436, 437, 540, 581 and 613. Significant associations with treatment outcome and parasite transmissibility have been shown for mutations at codons 437 [25] and 540 [19].

### Prevalence and post-treatment selection of mutant parasite genotypes in individuals included in transmission experiments

**Figure 1:**
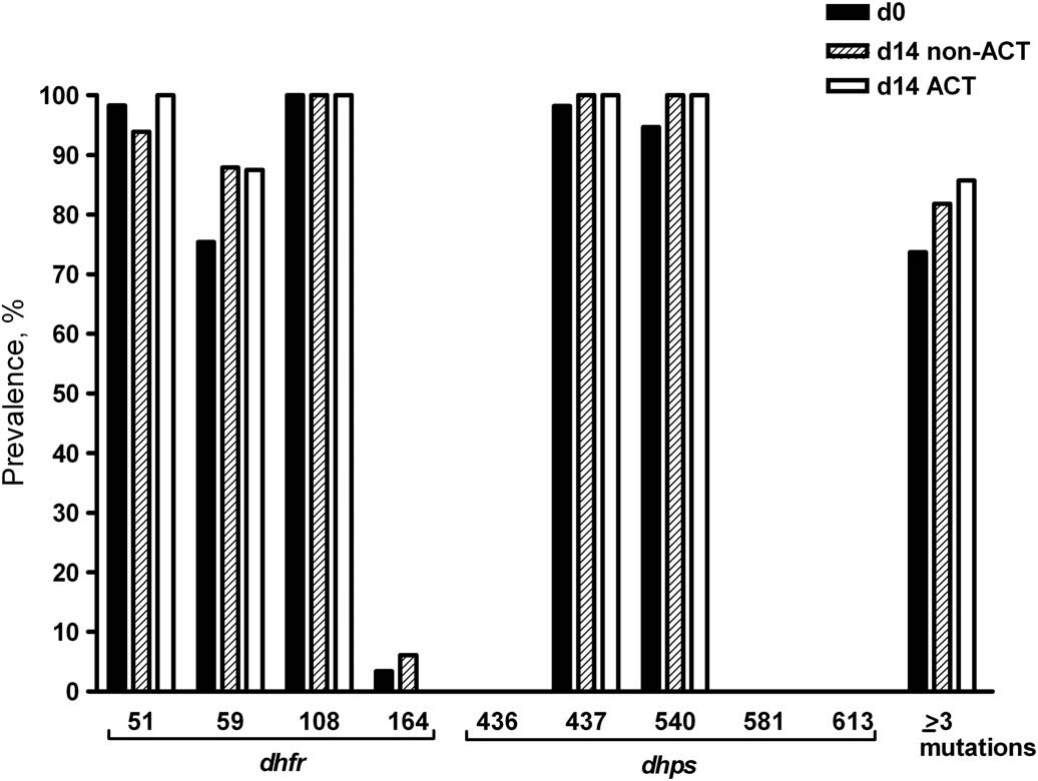
Prevalence of mutant genotypes for *dhfr* and *dhps* genes prior to treatment and on day 14 after non-ACT or ACT treatment in samples included in the membrane-feeding assays. Non-ACT = sulphadoxine-pyrimethamine (SP) alone or in combination with amodiaquine; ACT = SP in combination with artesunate; ≥ three mutations = mutations in c51+c59+c108 and possible additional mutations The number of observations on day 0: all mutations (n = 57–58) (see text); on day 14 after non-ACT treatment: c51, c59, c108, c164 (n = 33); c436, c437 (n = 23); c540, c581, c613 (n = 25); ≥ three mutations (n = 33); on day 14 after ACT treatment: c51, c59 (n = 8); c108, c164 (n = 7); c436, c437 (n = 4); c540, c581, c613 (n = 5); ≥ three mutations (n = 7).

Seventy-five individuals donated blood for random membrane-feeding experiments and subsequent QT-NASBA analyses [27]. Fifty-eight of these individuals contributed to parasite DNA analyses of SNPs in *dhfr* and *dhps* at both day 0 and day 14 (figure 1). In this group, *dhfr* genotyping for day 0 samples was successful in 100% (58/58) for all codons, except for c59 (57/58). For the day 14 samples, approximately half of whom were parasite-negative by microscopy, *dhfr* genotyping was successful in 71% (41/58) for c51 and c59, in 70% (40/58) for c108 and c164. *Dhps* genotyping for day 0 samples was successful in 98% (57/58) for all codons and for day 14 samples in 47% (27/58) for c436 and c437 and in 52% (30/58) for c540, c581 and c613. The 164L mutation was found in four infections, in one case as the majority (but mixed) genotype and in the other three cases as a mixed infection. The presence of the 164L mutation was verified by PCR-RFLP for all four samples.

DNA from 20 individuals in the SP+AQ treatment arm was available for *Pfcrt* genotyping. All 20 day 0 samples were successfully amplified, while only four of the 20 day 14 samples were amplified even after repeating the procedure. Nineteen of the twenty day 0 isolates harboured the mutant *Pfcrt* CVIET haplotype, and one individual harboured the CVMNK haplotype. Two of the four day 14 samples carried the CVIET haplotype and the other two the CVMNK haplotype. The SVMNT haplotype was not found. The remainder of the analyses will focus on *dhfr* and *dhps* mutations.

Fifteen of the 57 individuals (26.3%) included in the membrane-feeding experiments had an infection with two mutations in the *dhfr* gene at enrolment. The remaining 42 individuals (73.7%) had infections with three (n = 1), four (n = 2) or five (n = 39) mutations in the *dhfr* and *dhps* genes. Against the background of this high prevalence prior to treatment, mutations in c59 and c540 were more common in samples on day 14 after non-ACT treatment; although this was not statistically significant for either mutation (figure 1, McNemar test p = 0.375 and p = 1.00, respectively). The proportion of infections with ≥ three mutations did not increase significantly on day 14 after non-ACT treatment (McNemar test p = 0.687) or after ACT treatment (McNemar test p = 0.250).

### Mutant genotypes and malaria transmission

The median gametocyte density on day 14 after treatment was similar for both children harbouring infections with two mutations in the *dhfr* and *dhps* genes and for children with ≥ three mutations in these genes. We found no indication that infections with ≥ three mutations at enrolment were more transmissible (on day 14 after treatment) than infections presenting at day 0 with fewer mutations. The proportion of children infecting at least one mosquito and infected mosquitoes was similar for both groups (table 2). Infected mosquitoes (94/1710) harboured 1–13 oocysts. We observed no statistically significant association between the presence of ≥ three mutations and oocyst counts in mosquitoes for non-ACT treated individuals (β = 0.25, se(β) = 0.54, p = 0.64) or ACT treated individuals (β = 0.16, se(β) = 0.73, p = 0.82).

**Table 2:** Post-treatment malaria transmission in relation to mutations in the *dhfr* and *dhps* genes.

	*Double mutation* *	≥ *three mutations* *	*p-value*
Median gametocyte density/µL ¥ on day of feeding, (IQR)			
Non-ACT	10.40 (3.83–30.33)	4.96 (0.51–22.47)	0.27
ACT	0.84 (0.03–1.94)	0.17 (0.04–1.01)	0.66
Proportion of children infecting at least one mosquito, % (n/N)			
Non-ACT	77.8 (7/9)	73.5 (25/34)	1.00
ACT	50.0 (3/6)	62.5 (5/8)	1.00
Proportion of infected mosquitoes, % (n/N)§			
Non-ACT	7.0 (19/270)	6.0 (61/1020)	0.79
ACT	3.2 (6/180)	3.3 (8/240)	0.65

IQR = interquartile range; Non-ACT = treatment with sulphadoxine-pyrimethamine (SP) alone or in combination with amodiaquine; ACT = treatment with SP and artesunate (3 days).

¥ Gametocyte densities were measured by *Pfs25* QT-NASBA.

* Mutations were determined at enrolment: double mutation = mutations in c108+c59 or c108+c51; ≥ three mutations = mutations in c51+c59+c108 and possible additional mutations;

§ estimates were adjusted for correlation between observations from the same individual.

The comparison between ACT and non-ACT treated individuals was previously reported in detail for the entire dataset [27]. There, a significantly lower proportion of infected mosquitoes following ACT treatment was observed [27]. The current analyses show that this observed reduction in transmission potential is evident both for infections with two mutations in the *dhfr* and *dhps* genes and for those with ≥ three mutations (table 2). In the current selection of samples, ACT reduced the median gametocyte density on day of feeding (p<0.001), the proportion of infected mosquitoes (β = −0.86, se(β) = 0.46, p = 0.06) and the mean oocyst burden (β = −0.89, se(β) = 0.51, p = 0.08) compared to non-ACT treatment. However, we found no evidence this was linked to carriage of mutations in the *dhfr* and *dhps* genes, which was high in all treatment groups.

## Discussion

In this study, we observed a high prevalence of mutations in the *dhfr* and *dhps* genes of *P. falciparum* in an area in western Kenya. None of the infections harboured fewer than two mutations and the 164L mutation, which is rare in East Africa [16]–[19], [40], was found in four infections. Against this background of high prevalence rates of mutations in the *dhfr* and *dhps* genes, and the absence of wild-type infections to use as comparators, we found no association between the number of mutations in the *dhfr* or *dhps* genes and (sub)microscopic gametocytemia or the infectiousness to mosquitoes.

High prevalences of *dhfr* and *dhps* mutations are not uncommon in East Africa [15], [41], [42]. In our study population, where we previously reported that SP monotherapy gave an adequate clinical response in only 44% of the cases [27], we found that approximately 70% of the infections prior to treatment harboured *dhfr* loci with three mutations. The finding of the 164L mutation is an additional indication for the high level of SP resistance. There are only a few other studies that reported the 164L mutation in Africa [15]–[19]. In our study, three of the four samples with the 164L mutation also contained ≥ three mutations in other codons, indicating that the next step in the sequence of mutations has been reached. This suggests that SP treatment efficacy could deteriorate further [9], [13], [14] if drug pressure continues and strongly supports the policy change to AL as first-line antimalarial treatment [1]. Hamel and others found that the 164L mutation in a high endemic area was not associated with high-level SP resistance or poor outcome among adults [40], but the involvement of this mutation in treatment outcomes for children has yet to be elucidated.

We observed a high prevalence of the *Pfcrt* mutant CVIET haplotype, as previously observed in Kenya [43] and Tanzania [42], [44]. A persisting high prevalence of *Pfcrt* mutant parasites after abandoning CQ as first line treatment was also reported in central Kenya [45], although a reduction in its prevalence was observed in other countries [46], [47]. The *Pfcrt* gene is also associated with AQ resistance [44], [48]. AQ was widely used in the study area in government facilities in the period the study was conducted and continues to be a popular choice for home treatment with shop-bought drugs. In the year prior to the current study, we observed that 30.6% (26/85) of children attending the clinic with uncomplicated malaria had metabolites of 4-aminoquinolines in their urine (Bousema, unpublished observations). Although the used dipstick method could not differentiate between AQ and CQ, it is likely that the high frequency of the CVIET haplotype is currently being maintained by the use of AQ, rather than CQ.

Previous studies have found a relation between mutations in the *dhfr* and *dhps* genes and treatment outcome, but only when a substantial proportion of the parasite population harboured wild-type alleles [8], [21]. This was not the case in our population where we observed no additional risk of treatment failure for infections with ≥ three mutations in the *dhfr* gene compared to double mutations. In line with this, the number of parasite mutations was not related with the proportion of individuals in whom asexual parasites were still detectable on day 3, an indicator of parasite clearance time (PCT).

This is the first report that describes the relationship between submicroscopic gametocytemia and the presence of mutant genotypes. Mutations in the *dhfr* gene have previously been related to an increased microscopic gametocyte prevalence after SP treatment [20]–[23]. This observation may be partly explained by a longer PCT for mutant parasites; this prolonged survival time under drug pressure allows parasites to invest in transmission stages [20]. In an earlier study, we have shown that peaks in microscopic gametocyte prevalence after treatment are likely to be the result of an increase in the density of gametocytes that were already present before treatment at submicroscopic concentrations [49]. We therefore hypothesized that post-treatment gametocyte density (measured by QT-NASBA) would be increased for infections with mutations in the *dhfr* and *dhps* genes. However, the complete absence of wild-type infections in our study population prevented this comparison being made. When using infections with a double mutation in the *dhfr* gene as reference group, we did not observe any association between the presence of additional mutations in the *dhfr* or *dhps* genes and the prevalence or density of submicroscopic gametocytes. The transmission potential, quantified as the area under the curve of *Pfs25* QT-NASBA gametocyte density versus time, was not associated with additional mutations compared to the reference group. In line with this, we also observed no increased malaria transmission measured by membrane feeding experiments on day 14 after treatment in relation to additional *dhfr/dhps* mutations. This contrasts with studies from the Gambia where a substantial proportion of parasites carried wild-type *Pfcrt* alleles. Here, increased transmission of CQ resistant parasite strains was observed after examination of a similar number of transmission experiments and (infected) mosquitoes [23]–[25]. A recent study from Colombia also observed that infections with two *dhfr* mutations (in c108 and c51) had a 10-fold higher probability of infecting mosquitoes than infection with wild-type parasites [26].

### Limitations

In our study area of moderate transmission intensity, many of the study participants will have harboured multiple clone infections. We have presented estimates of mutation prevalence, and described infections as harbouring two, three or more mutations. However, in mixed infections individual mutations may be present on different parasite strains, and so it is not always possible to confidently infer which haplotypes are present.

None of the infections in our population consisted of pure wild-type parasites. This is likely to have obscured any relation between parasite resistance and malaria transmission. Our initial power calculations were based on assumptions derived from studies where wild-type samples were used as comparator group [23]–[25]. Using infections with a double mutation in the *dhfr* gene as comparator arm will have reduced our power to detect statistically significant associations, but allowed us to study the influence of additional mutations on transmission potential in an area where no infections contained fewer than two mutations in the *dhfr* gene. Barnes and colleagues recently showed a gradual increase in transmission potential with increasing number of mutations in the *dhfr* and *dhps* genes [22]. Despite the relatively small number of individuals with the quintuple mutation in that study (n = 17), the study detected a statistically significant 2.6-fold increased AUC compared to individuals with fewer mutations (n = 76) [22]. We did not observe a similar relation in our population.

### Overall evidence

Based on the available literature, it seems plausible that newly-arising resistance mutations result in a transmission advantage compared to wild-type parasites under drug pressure. However, in our study population, where the majority of infections harbour many resistance-associated mutations in the *dhfr* and *dhps* loci, we found no evidence that parasites with three or more mutations in the *dhfr* or *dhps* genes have an additional transmission advantage compared to double mutations. Our data also indicate that even at very high levels of SP resistance, the addition of AS to SP monotherapy can have a beneficial effect on malaria transmission.
